# Malignant Cardiac Tamponade: A Complication of Untreated Breast Cancer

**DOI:** 10.7759/cureus.26787

**Published:** 2022-07-12

**Authors:** Mohamed Ramzi Almajed, Mark S Obri, Wasih Kamran, Abigail Entz

**Affiliations:** 1 Internal Medicine, Henry Ford Hospital, Detroit, USA; 2 Radiology, Henry Ford Hospital, Detroit, USA

**Keywords:** social determinants of health, palliative medicine, pericardial window, pericardiocentesis, breast cancer, malignant pericardial effusion, carcinomatous pericarditis, cardiac tamponade

## Abstract

Carcinomatous pericarditis is a rare complication of locally aggressive breast cancer in which malignant cells directly extend into the pericardium causing inflammation and creating a pericardial effusion. A 40-year-old woman with untreated metastatic breast cancer presented to an outpatient clinic in significant distress with symptoms of progressive shortness of breath and bilateral leg swelling. An urgent echocardiogram demonstrated a large pericardial effusion with echocardiographic evidence of cardiac tamponade. She underwent emergent pericardiocentesis of the effusion that was deemed to be malignant after cytologic evaluation. Subsequently, she opted for palliative treatment involving the surgical creation of a right pericardial window and placement of an indwelling pleural catheter. Internists should maintain a high index of suspicion for malignant cardiac tamponade in at-risk patients, especially those with locally aggressive and advanced malignancies.

## Introduction

Carcinomatous pericarditis is inflammation of the pericardium secondary to direct extension of malignant cells; this inflammation can result in the accumulation of an exudative fluid that creates a pericardial effusion. Progressive accumulation of fluid in the pericardial space increases the pericardial pressure, decreases venous return, and impairs cardiac output in a phenomenon known as tamponade physiology. Subacute cardiac tamponade describes the development of tamponade physiology over several weeks or months and is frequently seen in the setting of carcinomatous pericarditis [1].

Secondary, or metastatic, neoplasms more commonly involve the pericardium than primary neoplasms. The most common primary sites of metastatic involvement include lung carcinomas, breast carcinomas, hematological malignancies, and gastrointestinal carcinomas [2]. Malignant involvement of the pericardium has been identified in 5% to 20% of patients with metastatic neoplasms; however, the clinically significant pericardial disease is considerably less common [3]. In an autopsy series of 1029 patients diagnosed with malignant neoplasms, 110 (10.7%) were found to have cardiac involvement and of these patients, 28 (25.4%) developed pericardial effusions [4]. Pericardial effusions due to malignancy are typically larger and associated with worse outcomes compared to non-malignant effusions. Cardiac tamponade may occur in up to 50% of patients with malignant pericardial effusions [5].

We present a case of a young woman with metastatic and locally invasive breast carcinoma who opted against treatment and presented with malignant cardiac tamponade.

## Case presentation

A 40-year-old African American woman presented to the outpatient clinic with complaints of progressive shortness of breath for two weeks. Her symptoms were worse on exertion and in the supine position, whereas leaning forward improved her symptoms. She had swelling in both legs but did not have chest pain, cough, sputum production, or fever.

Her medical history was significant for invasive ductal carcinoma of the right breast that was locally aggressive with lymphatic and osseous metastases. Her illness was complicated by recurrent right-sided malignant pleural effusions that required routine thoracentesis. She received her cancer diagnosis two years prior and was recommended for surgery, chemotherapy, and radiation. However, she declined treatment as it did not align with her health beliefs.

**Figure 1:**
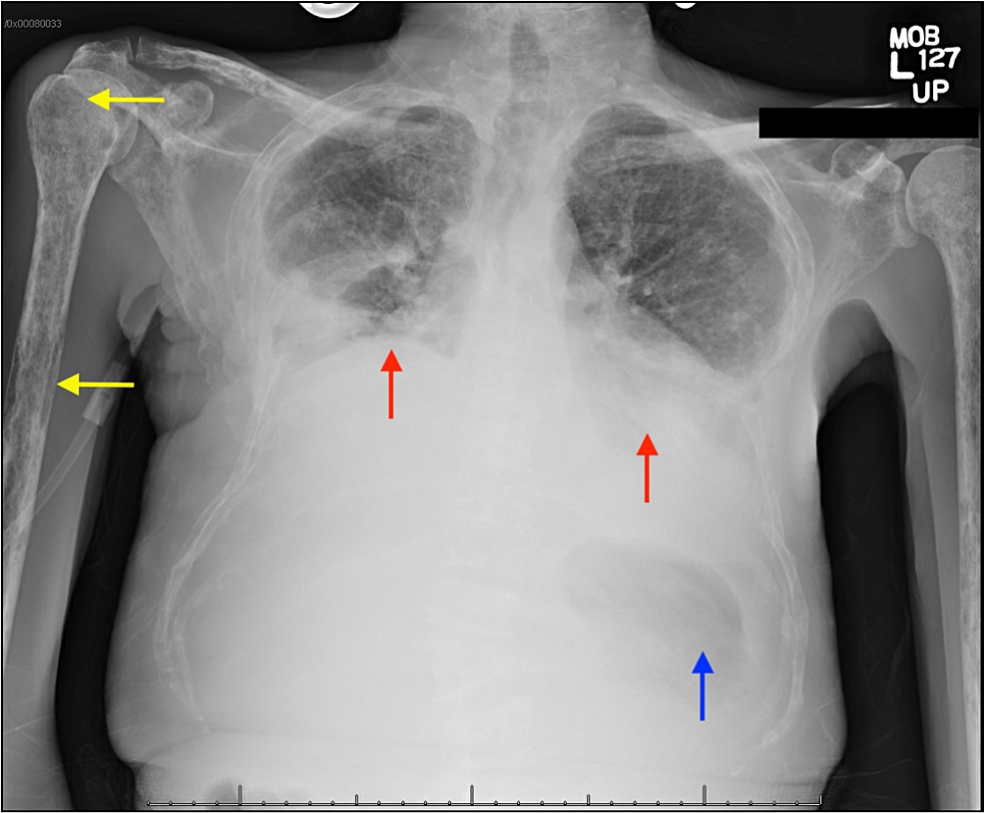
Chest x-ray in an upright antero-posterior view demonstrating bilateral pleural effusions (red arrows), an obscured cardiac silhouette (blue arrow), and diffuse osseous lytic lesions (yellow arrows).

In the clinic, her vital signs were remarkable for tachycardia with a heart rate of 133 beats per minute and tachypnea with a respiratory rate of 23 breaths per minute; she was not hypoxic and had an oxygen saturation of 97% on room air. Her examination was remarkable for labored breathing, decreased bibasilar breath sounds, and distant heart sounds. Within the month prior to presentation, the patient had been seen in several different clinical settings and reported similar symptoms that were attributed to her progressive malignancy and known pleural effusions. She underwent two thoracenteses with minimal improvement in her symptoms, and a recent x-ray of the chest showed bilateral pleural effusions and an obscured cardiac silhouette (Figure 1). Given her persistent symptoms in the setting of known untreated breast cancer, alternative diagnoses were considered at this time, which included cardiac tamponade, pericardial effusion, pulmonary embolism, and heart failure.

**Figure 2:**
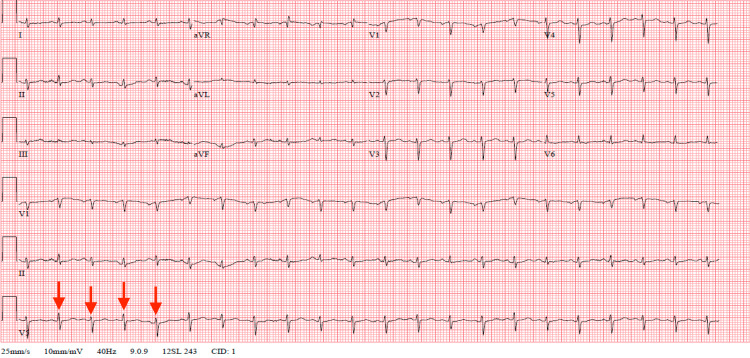
Electrocardiogram remarkable for sinus tachycardia, low-voltage QRS complexes, and electrical alternans (red arrows).

**Figure 3:**
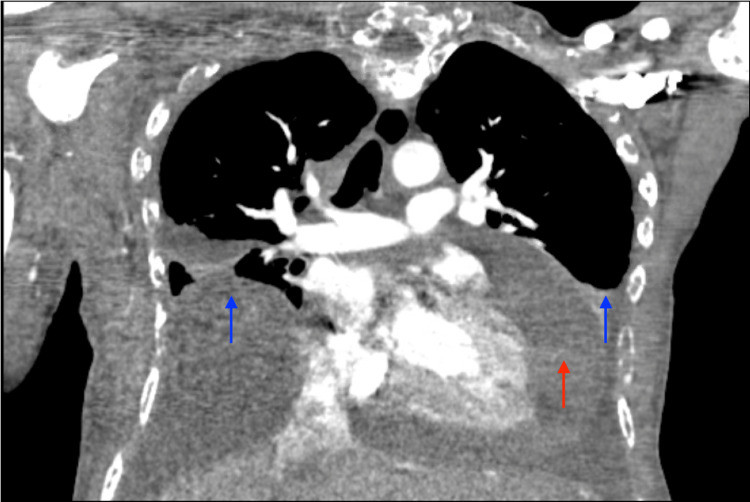
CT scan of the chest in coronal view demonstrates a large pericardial effusion (red arrow) and bilateral pleural effusions (blue arrows).

**Figure 4:**
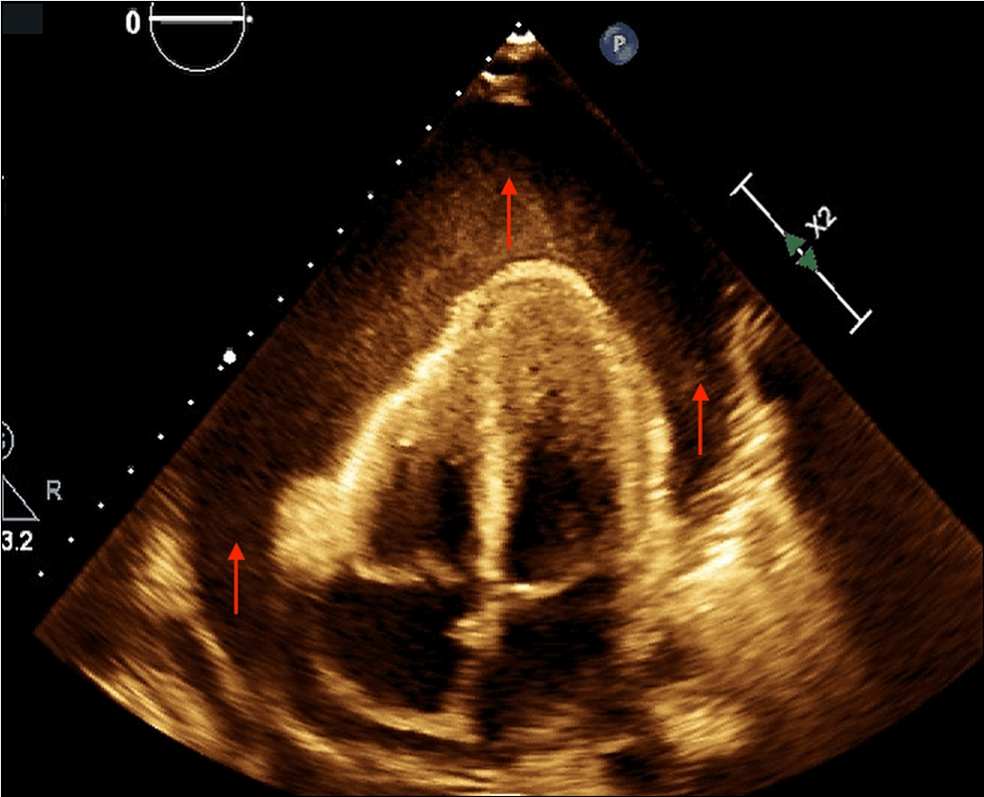
Echocardiogram in an apical four-chamber view shows a large pericardial effusion present circumferentially around the entire heart (red arrows).

Her workup was remarkable for an electrocardiogram that demonstrated electrical alternans (Figure 2). A CT scan of the chest showed a large pericardial effusion without signs of pulmonary embolism (Figure 3). An urgent echocardiogram revealed a large pericardial effusion present circumferentially around the entire heart with the pathognomonic appearance of a “swinging heart”. Echocardiographic evidence of cardiac tamponade was seen with systolic and diastolic right ventricular wall collapse (Figure 4).

**Figure 5:**
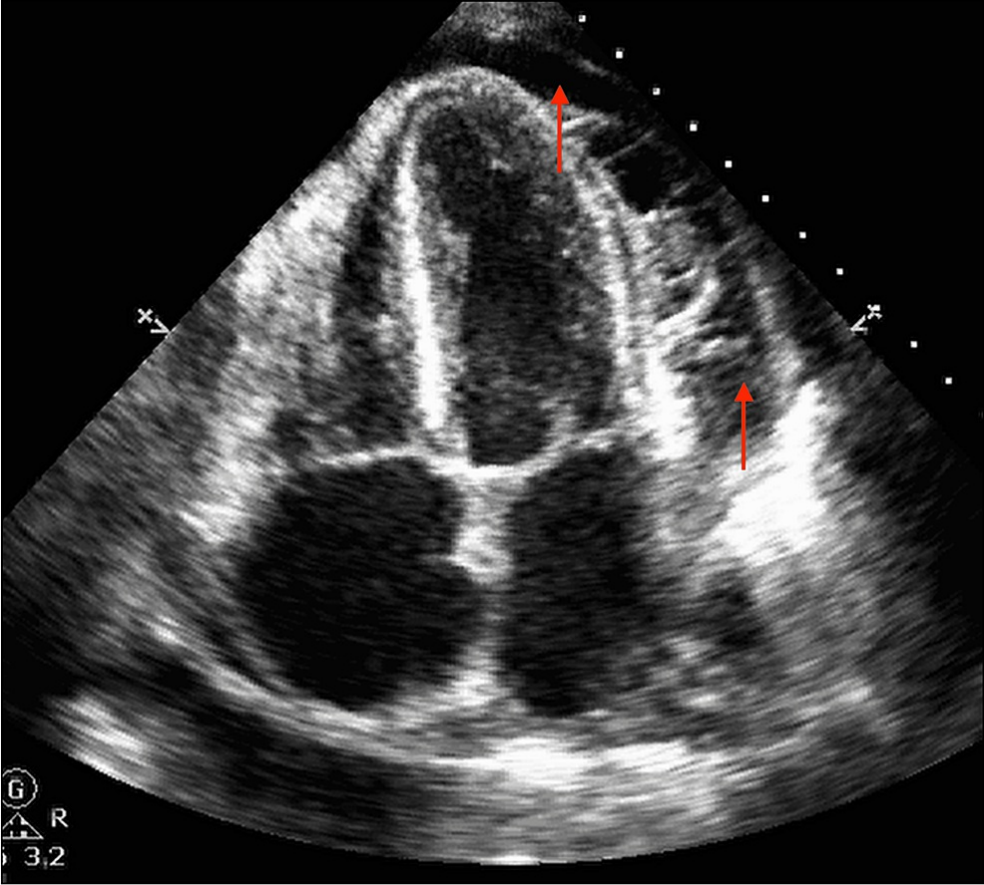
Echocardiogram in an apical four-chamber view showing a decrease in the size of the pericardial effusion after pericardiocentesis (red arrows).

The patient underwent emergent pericardiocentesis with the drainage of 800 ml of the bloody fluid. The cytologic evaluation was positive for malignant cells with a diagnosis of metastatic adenocarcinoma consistent with a breast primary neoplasm. She experienced significant improvement in her symptoms after the procedure; however, the effusion re-accumulated within days (Figure 5). After extensive goals of care discussions, she elected for palliative treatment with surgical creation of a right pericardial window and placement of an indwelling pleural catheter.

## Discussion

Pericardial effusion associated with a malignancy confers a poor prognosis as it represents the extension of malignant cells into the pericardium, a phenomenon that occurs in the setting of invasive local neoplasms or metastatic neoplastic spread. Several large retrospective studies determined a mean survival of four to six months from the diagnosis of malignant pericardial effusion in patients with both previously known or newly identified malignancies [6-8]. However, in about two-thirds of patients diagnosed with cancer, pericardial effusion is caused by non-malignant processes such as chemotherapy, radiation therapy, and infections [9]. Pericardial fluid cytology that is positive for neoplastic cells is an independent poor prognostic factor [10,11].

Cardiac tamponade is a pericardial compression syndrome with a state of low cardiac output that occurs as a consequence of the accumulation of fluid in the pericardial space. The pericardium's elasticity initially accommodates the increase in volume and pressure; however, the progressive increase in fluid eventually overcomes the pericardium's elastic limit causing external pressure on the cardiac chambers. Increased pressure in the pericardial space compresses the cardiac chambers, decreases venous return to the heart, and impairs diastolic filling [12]. Since the volume of the cardiac chambers decreases, less blood fills the chambers and a lower volume is ejected with each contraction; this decrease in stroke volume results in lower cardiac output and blood pressure. Clinical features of tamponade are manifestations of impaired venous return to the heart and decreased systemic perfusion. In the early stages, patients experience dyspnea, chest discomfort and pain, and fatigue. After tamponade physiology manifests, patients deteriorate and develop a state of shock [13].

Management options for malignant pericardial effusions are limited and rarely curative. In patients presenting with cardiac tamponade, emergent pericardiocentesis is indicated to prevent shock and death [14]. Initial management of large effusions without tamponade is with diagnostic and therapeutic pericardiocentesis. Analysis of the pericardial fluid establishes the neoplastic nature of the effusion and delays recurrence and hemodynamic compromise [5]. Definitive oncological treatment of the underlying malignancy improves outcomes, especially if the effusion is associated with direct extension of a locally invasive neoplasm [14,15]. Recurrence prevention can be achieved by routine pericardiocentesis, intrapericardial injection of cytostatic and sclerosing agents, or surgical creation of a pericardial window [16-18].

The advent of screening guidelines and advanced imaging modalities for breast cancer has resulted in earlier identification of the malignancy along its disease process, which has made curative therapy attainable for more patients. Patients in whom the underlying malignancy is not managed in a timely and appropriate manner could develop more severe complications and have worse outcomes. In this case, an amalgam of societal, environmental, and political factors prevented our patient from pursuing treatment for breast cancer; the malignancy remained untreated, invaded local tissues, infiltrated the pericardium, and ultimately resulted in cardiac tamponade. She had initially been diagnosed with early-stage breast cancer and was offered definitive surgery, which she declined, despite receiving similar recommendations from three cancer institutes over the span of a year. The patient demonstrated insight into her condition and had appropriate decision-making capacity. However, she was hesitant to pursue treatment as she was uncertain of its applicability to young African American women such as herself. After her breast cancer became metastatic, the patient was re-evaluated and offered chemotherapy and radiation therapy, which she declined as well. Social determinants of health are a key factor for our patient’s poor outcome and avoidable complications as her health beliefs resulted in her concluding that no appropriate treatments were available.

Preventing the recurrence of pericardial effusion is aimed at the relief of symptoms rather than the treatment of the underlying disease. Interventions such as the creation of a surgical pericardial window are palliative as they allow for improvement in quality of life and a reduction in hospitalizations for patients with advanced malignancies. This aligned with the values and goals of our patient as she experienced relief from her shortness of breath, increased functional status, and greater independence in her activities of daily living. After discharge, she continued to follow-up with her outpatient physicians and expressed content and satisfaction with her palliative treatment.

## Conclusions

Internists should maintain a high index of suspicion for malignant cardiac tamponade in at-risk patients. The most commonly implicated malignancies include lung carcinoma, breast carcinoma, hematological malignancies, and gastrointestinal carcinoma. Pericardiocentesis is indicated emergently in large effusions with tamponade; it is also indicated as a diagnostic and therapeutic intervention for large effusions without tamponade. Although there is no definitive treatment, management is generally palliative to prevent progression to cardiac tamponade and to improve quality of life by mitigating symptoms.

## References

[1] Seferović PM, Ristić AD, Maksimović R (2013). Pericardial syndromes: an update after the ESC guidelines 2004. Heart Fail Rev.

[2] Goldberg AD, Blankstein R, Padera RF (2013). Tumors metastatic to the heart. Circulation.

[3] Maisch B, Ristic A, Pankuweit S (2010). Evaluation and management of pericardial effusion in patients with neoplastic disease. Prog Cardiovasc Dis.

[4] Klatt EC, Heitz DR (1990). Cardiac metastases. Cancer.

[5] Imazio M, Colopi M, De Ferrari GM (2020). Pericardial diseases in patients with cancer: contemporary prevalence, management and outcomes. Heart.

[6] Wagner PL, McAleer E, Stillwell E, Bott M, Rusch VW, Schaffer W, Huang J (2011). Pericardial effusions in the cancer population: prognostic factors after pericardial window and the impact of paradoxical hemodynamic instability. J Thorac Cardiovasc Surg.

[7] Kim SH, Kwak MH, Park S (2010). Clinical characteristics of malignant pericardial effusion associated with recurrence and survival. Cancer Res Treat.

[8] Ben-Horin S, Bank I, Guetta V, Livneh A (2006). Large symptomatic pericardial effusion as the presentation of unrecognized cancer: a study in 173 consecutive patients undergoing pericardiocentesis. Medicine (Baltimore).

[9] Vaitkus PT, Herrmann HC, LeWinter MM (1994). Treatment of malignant pericardial effusion. JAMA.

[10] Gornik HL, Gerhard-Herman M, Beckman JA (2005). Abnormal cytology predicts poor prognosis in cancer patients with pericardial effusion. J Clin Oncol.

[11] Dequanter D, Lothaire P, Berghmans T, Sculier JP (2008). Severe pericardial effusion in patients with concurrent malignancy: a retrospective analysis of prognostic factors influencing survival. Ann Surg Oncol.

[12] Reddy PS, Curtiss EI, Uretsky BF (1990). Spectrum of hemodynamic changes in cardiac tamponade. Am J Cardiol.

[13] LeWinter MM (2014). Acute pericarditis. N Engl J Med.

[14] Adler Y, Charron P, Imazio M (2015). 2015 ESC guidelines for the diagnosis and management of pericardial diseases: The Task Force for the Diagnosis and Management of Pericardial Diseases of the European Society of Cardiology (ESC) Endorsed by: The European Association for Cardio-Thoracic Surgery (EACTS). Eur Heart J.

[15] Apodaca-Cruz A, Villarreal-Garza C, Torres-Avila B (2010). Effectiveness and prognosis of initial pericardiocentesis in the primary management of malignant pericardial effusion. Interact Cardiovasc Thorac Surg.

[16] Bischiniotis TS, Lafaras CT, Platogiannis DN (2005). Intrapericardial cisplatin administration after pericardiocentesis in patients with lung adenocarcinoma and malignant cardiac tamponade. Hellenic J Cardiol.

[17] Patel N, Rafique AM, Eshaghian S, Mendoza F, Biner S, Cercek B, Siegel RJ (2013). Retrospective comparison of outcomes, diagnostic value, and complications of percutaneous prolonged drainage versus surgical pericardiotomy of pericardial effusion associated with malignancy. Am J Cardiol.

[18] Martinoni A, Cipolla CM, Civelli M (2000). Intrapericardial treatment of neoplastic pericardial effusions. Herz.

